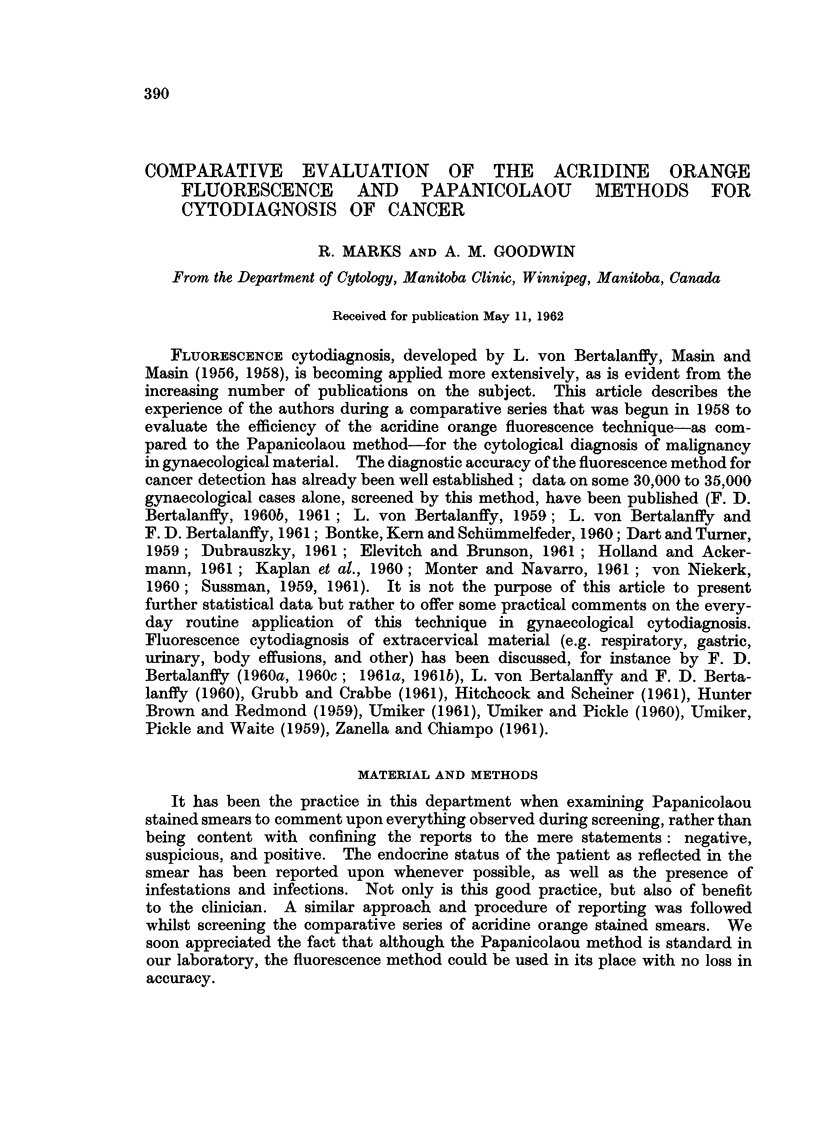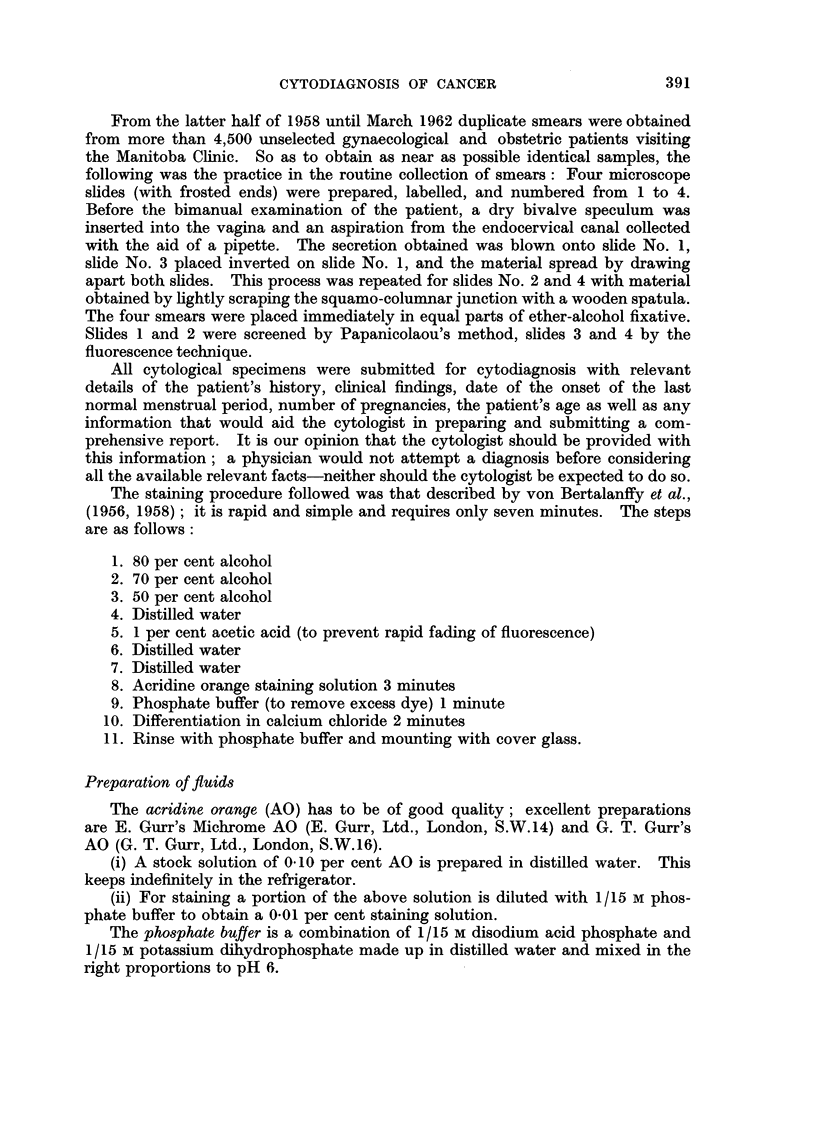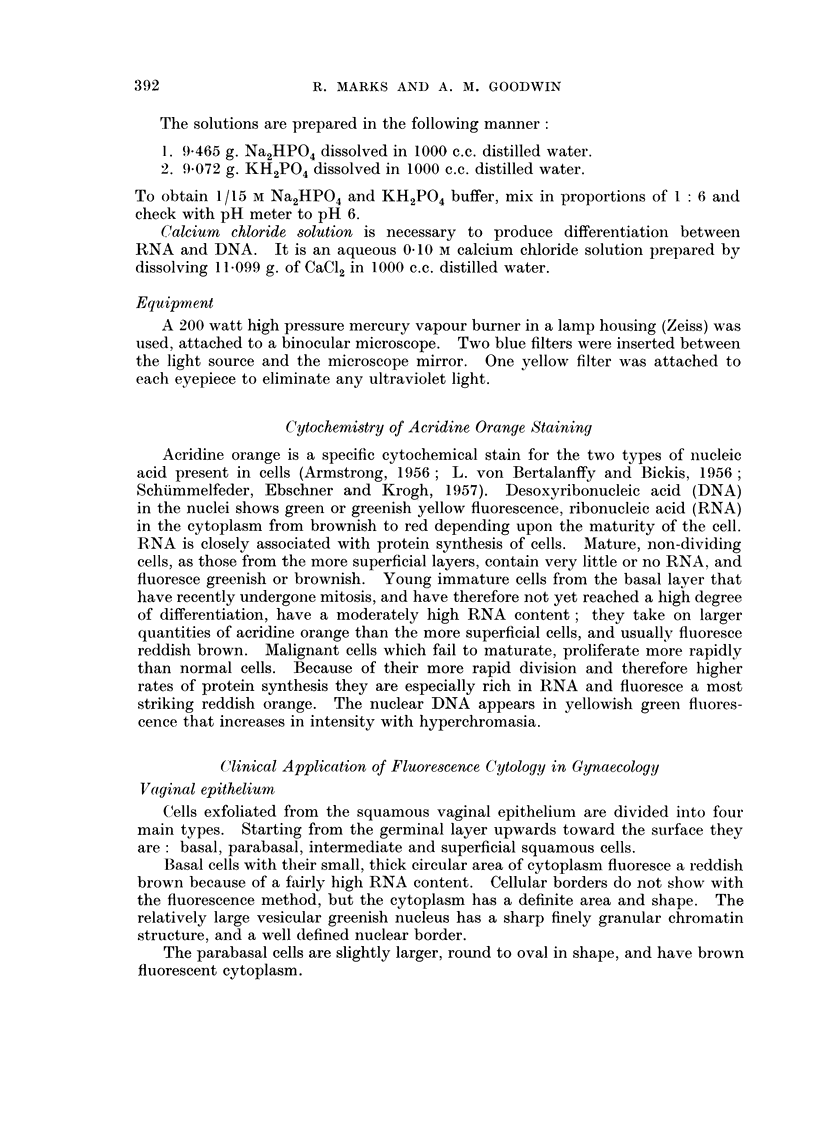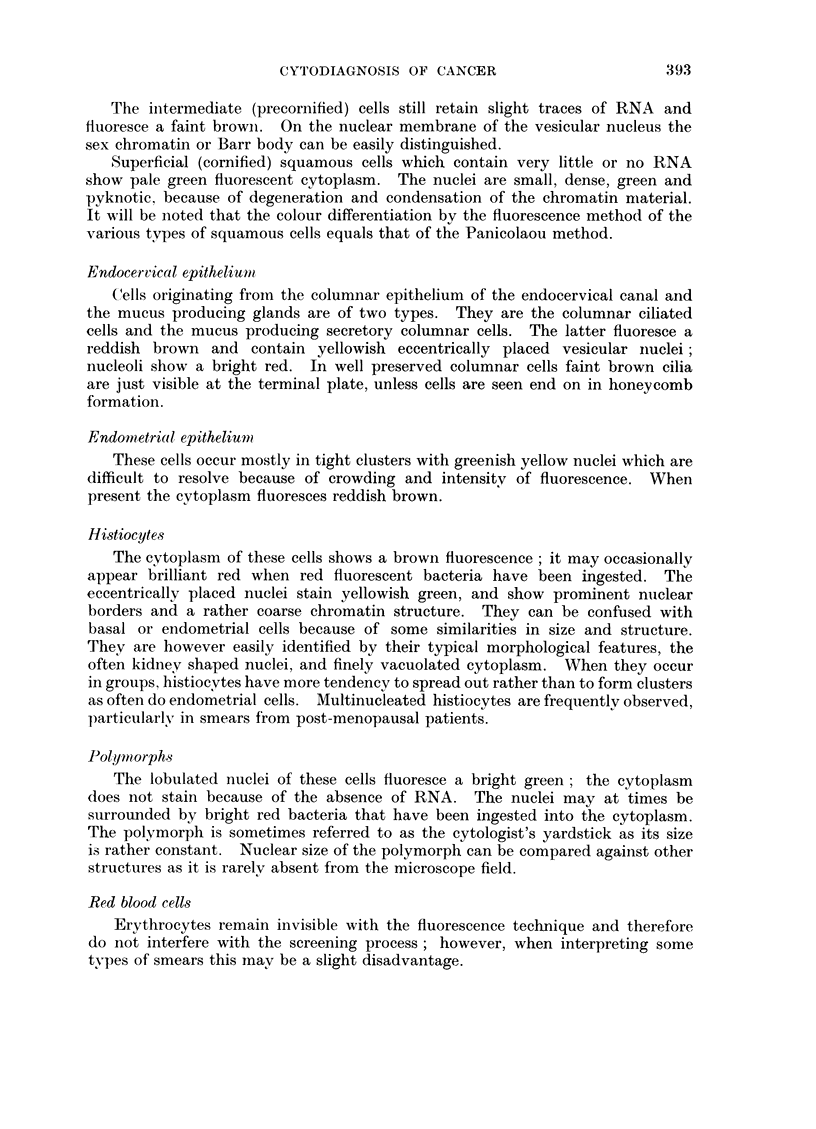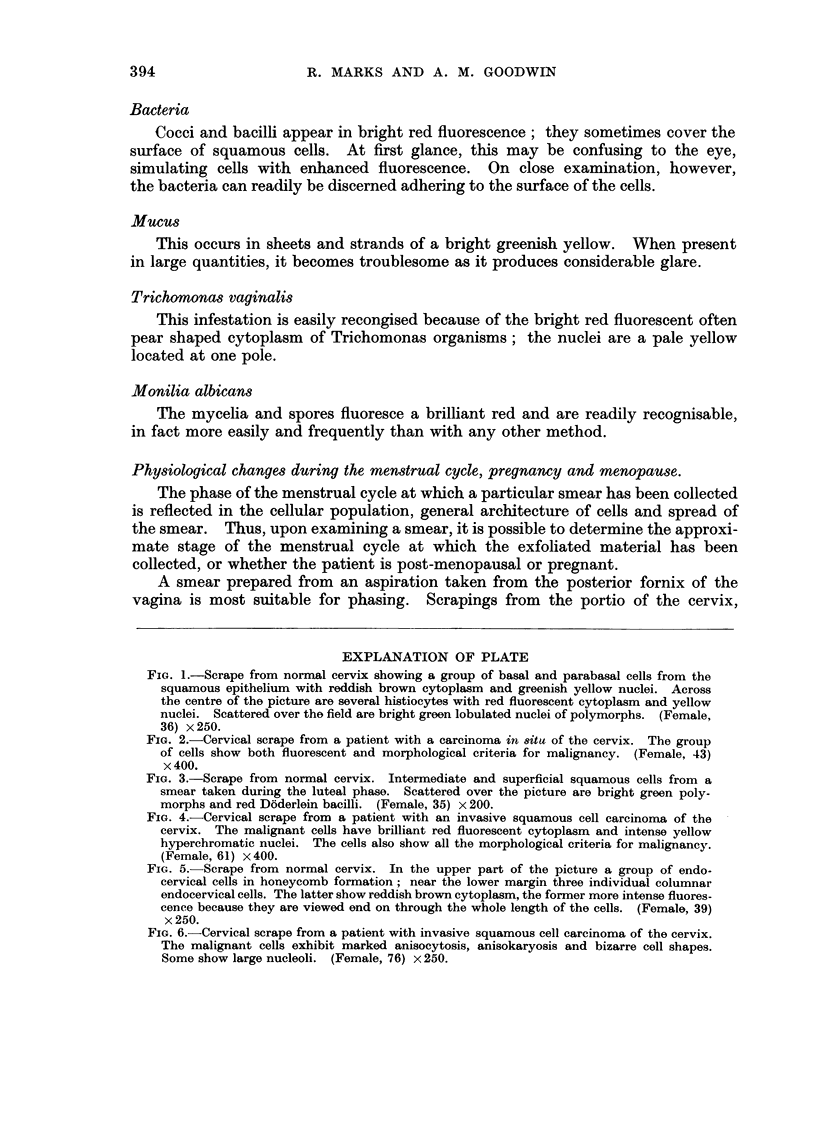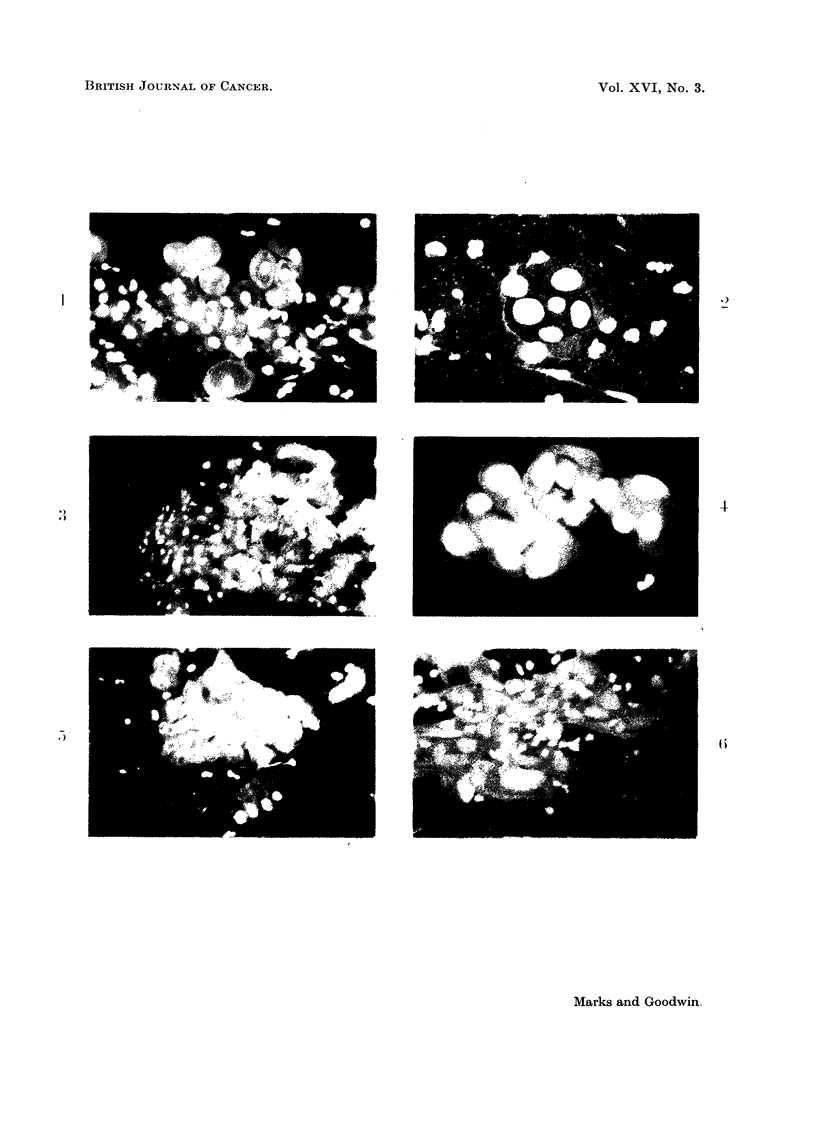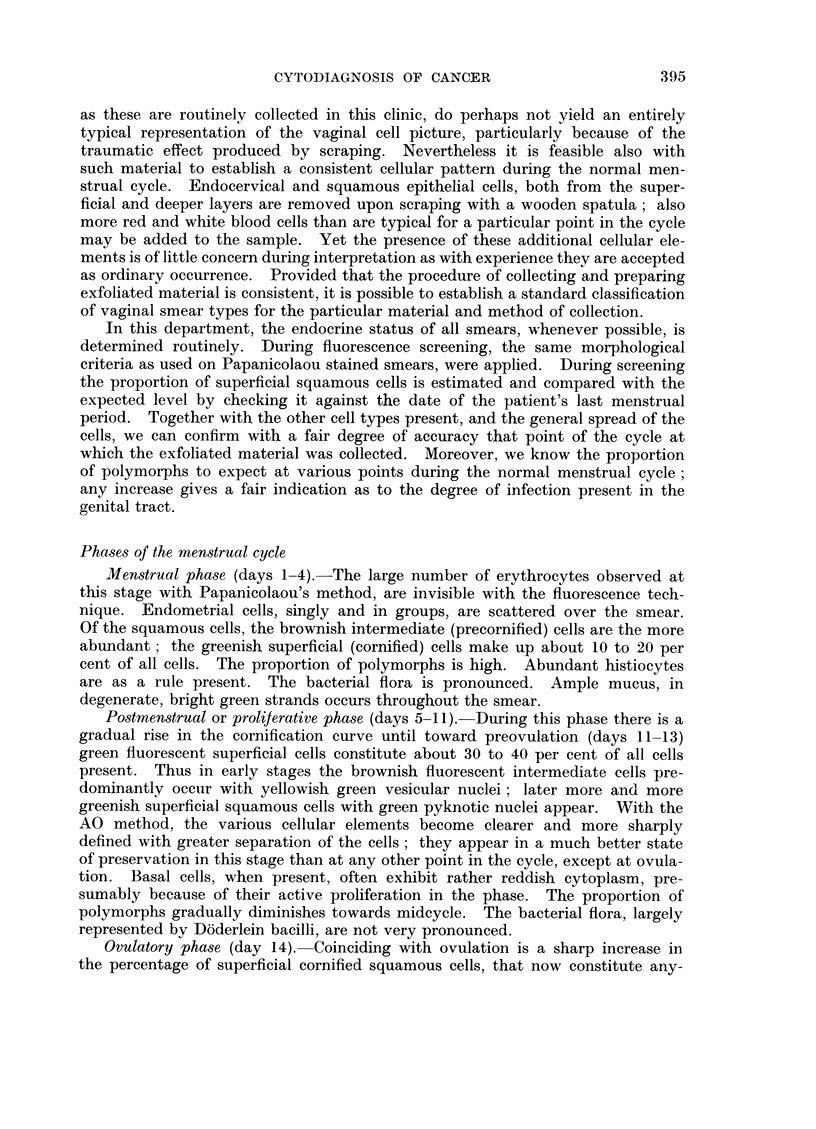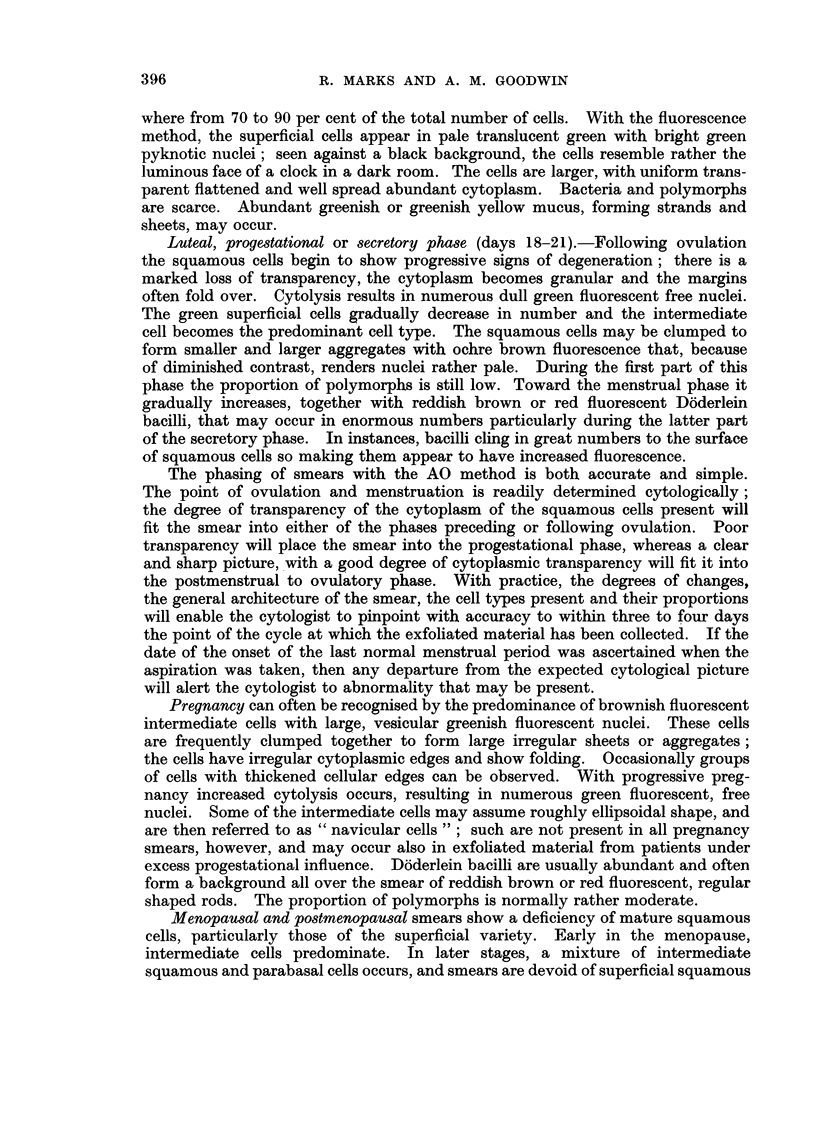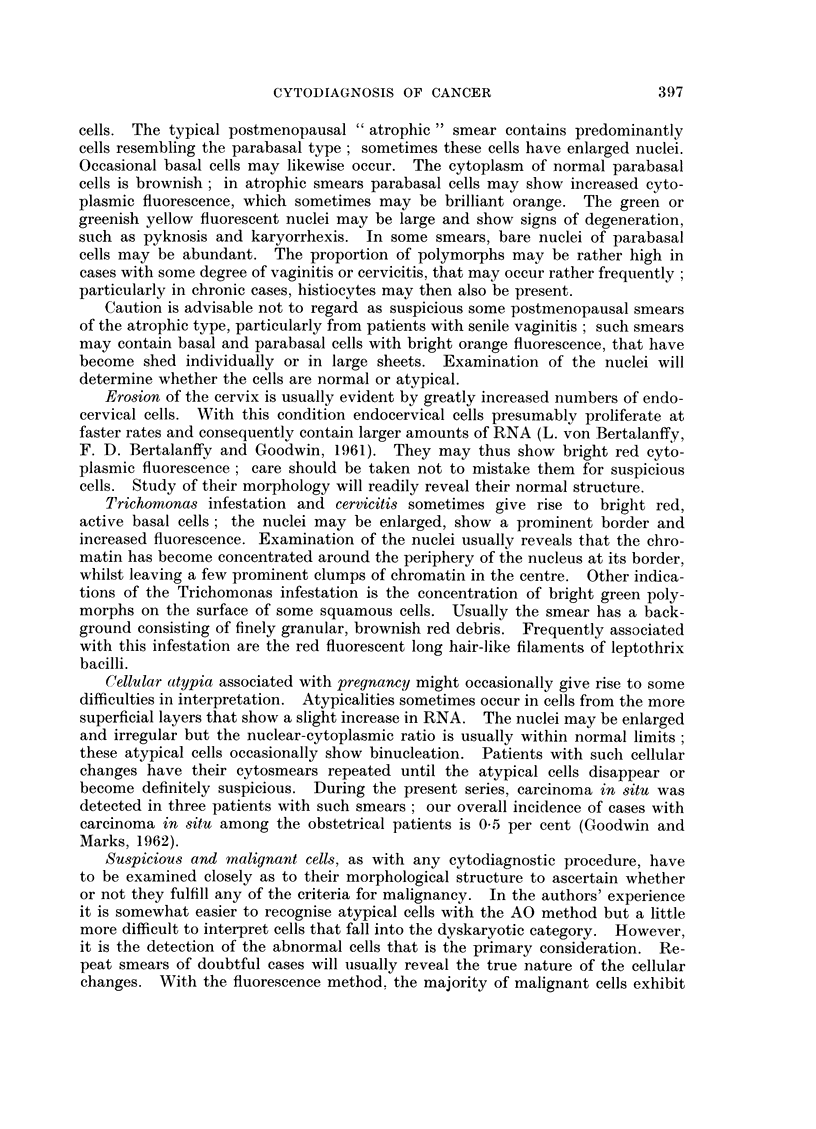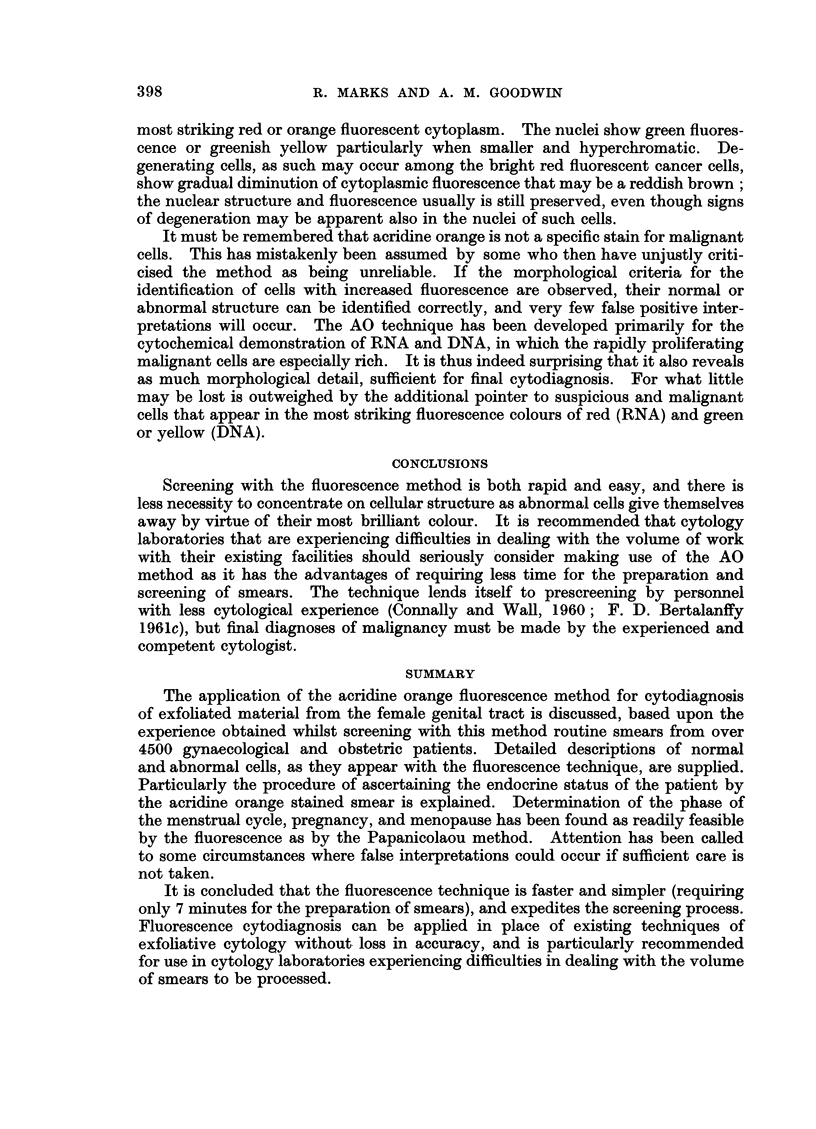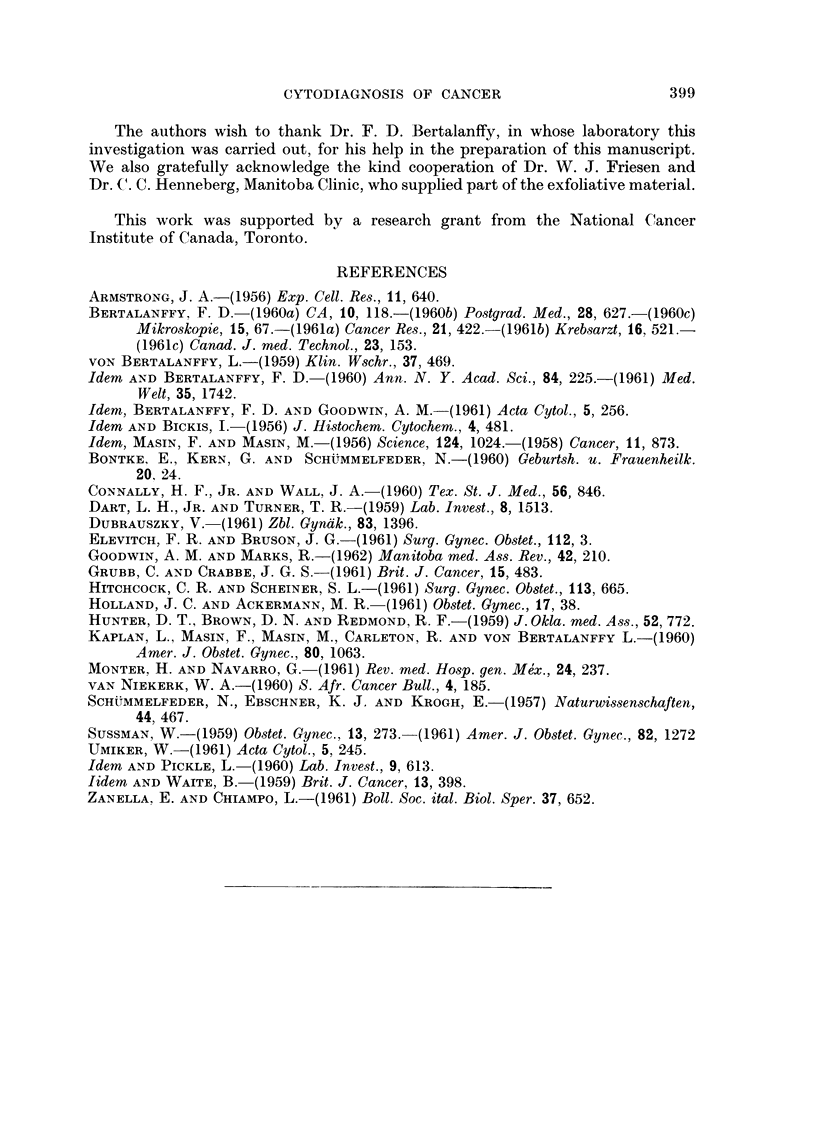# Comparative Evaluation of the Acridine Orange Fluorescence and Papanicolaou Methods for Cytodiagnosis of Cancer

**DOI:** 10.1038/bjc.1962.45

**Published:** 1962-09

**Authors:** R. Marks, A. M. Goodwin

## Abstract

**Images:**


### COMPARATIVE EVALUATION OF THE ACRIDINE ORANGE FLUORESCENCE AND PAPANICOLAOU METHODS FOR CYTODIAGNOSIS OF CANCER

R. MARKS AND A. M. GOODWIN

*From the Department of Cytology, Manitoba Clinic, Winnipeg, Manitoba, Canada*

Received for publication May 11, 1962

FLUORESCENCE cytodiagnosis, developed by L. von Bertalanffy, Masin and Masin (1956, 1958), is becoming applied more extensively, as is evident from the increasing number of publications on the subject. This article describes the experience of the authors during a comparative series that was begun in 1958 to evaluate the efficiency of the acridine orange fluorescence technique—as compared to the Papanicolaou method—for the cytological diagnosis of malignancy in gynaecological material. The diagnostic accuracy of the fluorescence method for cancer detection has already been well established ; data on some 30,000 to 35,000 gynaecological cases alone, screened by this method, have been published (F. D. Bertalanffy, 1960*b*, 1961 ; L. von Bertalanffy, 1959 ; L. von Bertalanffy and F. D. Bertalanffy, 1961 ; Bontke, Kern and Schümmelfeder, 1960 ; Dart and Turner, 1959 ; Dubrauszky, 1961 ; Elevitch and Brunson, 1961 ; Holland and Ackermann, 1961 ; Kaplan *et al.*, 1960 ; Monter and Navarro, 1961 ; von Niekerk, 1960 ; Sussman, 1959, 1961). It is not the purpose of this article to present further statistical data but rather to offer some practical comments on the everyday routine application of this technique in gynaecological cytodiagnosis. Fluorescence cytodiagnosis of extracervical material (e.g. respiratory, gastric, urinary, body effusions, and other) has been discussed, for instance by F. D. Bertalanffy (1960*a*, 1960*c* ; 1961*a*, 1961*b*), L. von Bertalanffy and F. D. Bertalanffy (1960), Grubb and Crabbe (1961), Hitchcock and Scheiner (1961), Hunter Brown and Redmond (1959), Umiker (1961), Umiker and Pickle (1960), Umiker, Pickle and Waite (1959), Zanella and Chiampo (1961).

### MATERIAL AND METHODS

It has been the practice in this department when examining Papanicolaou stained smears to comment upon everything observed during screening, rather than being content with confining the reports to the mere statements : negative, suspicious, and positive. The endocrine status of the patient as reflected in the smear has been reported upon whenever possible, as well as the presence of infestations and infections. Not only is this good practice, but also of benefit to the clinician. A similar approach and procedure of reporting was followed whilst screening the comparative series of acridine orange stained smears. We soon appreciated the fact that although the Papanicolaou method is standard in our laboratory, the fluorescence method could be used in its place with no loss in accuracy.

From the latter half of 1958 until March 1962 duplicate smears were obtained from more than 4,500 unselected gynaecological and obstetric patients visiting the Manitoba Clinic. So as to obtain as near as possible identical samples, the following was the practice in the routine collection of smears : Four microscope slides (with frosted ends) were prepared, labelled, and numbered from 1 to 4. Before the bimanual examination of the patient, a dry bivalve speculum was inserted into the vagina and an aspiration from the endocervical canal collected with the aid of a pipette. The secretion obtained was blown onto slide No. 1, slide No. 3 placed inverted on slide No. 1, and the material spread by drawing apart both slides. This process was repeated for slides No. 2 and 4 with material obtained by lightly scraping the squamo-columnar junction with a wooden spatula. The four smears were placed immediately in equal parts of ether-alcohol fixative. Slides 1 and 2 were screened by Papanicolaou's method, slides 3 and 4 by the fluorescence technique.

All cytological specimens were submitted for cytodiagnosis with relevant details of the patient's history, clinical findings, date of the onset of the last normal menstrual period, number of pregnancies, the patient's age as well as any information that would aid the cytologist in preparing and submitting a comprehensive report. It is our opinion that the cytologist should be provided with this information ; a physician would not attempt a diagnosis before considering all the available relevant facts—neither should the cytologist be expected to do so.

The staining procedure followed was that described by von Bertalanffy *et al.*, (1956, 1958) ; it is rapid and simple and requires only seven minutes. The steps are as follows :

1. 80 per cent alcohol
2. 70 per cent alcohol
3. 50 per cent alcohol
4. Distilled water
5. 1 per cent acetic acid (to prevent rapid fading of fluorescence)
6. Distilled water
7. Distilled water
8. Acridine orange staining solution 3 minutes
9. Phosphate buffer (to remove excess dye) 1 minute
10. Differentiation in calcium chloride 2 minutes
11. Rinse with phosphate buffer and mounting with cover glass.

*Preparation of fluids*

The *acridine orange* (AO) has to be of good quality ; excellent preparations are E. Gurr's Michrome AO (E. Gurr, Ltd., London, S.W.14) and G. T. Gurr's AO (G. T. Gurr, Ltd., London, S.W.16).

(i) A stock solution of 0·10 per cent AO is prepared in distilled water. This keeps indefinitely in the refrigerator.

(ii) For staining a portion of the above solution is diluted with 1/15 M phosphate buffer to obtain a 0·01 per cent staining solution.

The *phosphate buffer* is a combination of 1/15 M disodium acid phosphate and 1/15 M potassium dihydrophosphate made up in distilled water and mixed in the right proportions to pH 6.

The solutions are prepared in the following manner :

1. 9·465 g. $Na_2HPO_4$ dissolved in 1000 c.c. distilled water.
2. 9·072 g. $KH_2PO_4$ dissolved in 1000 c.c. distilled water.

To obtain 1/15 M $Na_2HPO_4$ and $KH_2PO_4$ buffer, mix in proportions of 1 : 6 and check with pH meter to pH 6.

*Calcium chloride solution* is necessary to produce differentiation between RNA and DNA. It is an aqueous 0·10 M calcium chloride solution prepared by dissolving 11·099 g. of $CaCl_2$ in 1000 c.c. distilled water.

*Equipment*

A 200 watt high pressure mercury vapour burner in a lamp housing (Zeiss) was used, attached to a binocular microscope. Two blue filters were inserted between the light source and the microscope mirror. One yellow filter was attached to each eyepiece to eliminate any ultraviolet light.

### *Cytochemistry of Acridine Orange Staining*

Acridine orange is a specific cytochemical stain for the two types of nucleic acid present in cells (Armstrong, 1956 ; L. von Bertalanffy and Bickis, 1956 ; Schümmelfeder, Ebschner and Krogh, 1957). Desoxyribonucleic acid (DNA) in the nuclei shows green or greenish yellow fluorescence, ribonucleic acid (RNA) in the cytoplasm from brownish to red depending upon the maturity of the cell. RNA is closely associated with protein synthesis of cells. Mature, non-dividing cells, as those from the more superficial layers, contain very little or no RNA, and fluoresce greenish or brownish. Young immature cells from the basal layer that have recently undergone mitosis, and have therefore not yet reached a high degree of differentiation, have a moderately high RNA content ; they take on larger quantities of acridine orange than the more superficial cells, and usually fluoresce reddish brown. Malignant cells which fail to maturate, proliferate more rapidly than normal cells. Because of their more rapid division and therefore higher rates of protein synthesis they are especially rich in RNA and fluoresce a most striking reddish orange. The nuclear DNA appears in yellowish green fluorescence that increases in intensity with hyperchromasia.

### *Clinical Application of Fluorescence Cytology in Gynaecology*

*Vaginal epithelium*

Cells exfoliated from the squamous vaginal epithelium are divided into four main types. Starting from the germinal layer upwards toward the surface they are : basal, parabasal, intermediate and superficial squamous cells.

Basal cells with their small, thick circular area of cytoplasm fluoresce a reddish brown because of a fairly high RNA content. Cellular borders do not show with the fluorescence method, but the cytoplasm has a definite area and shape. The relatively large vesicular greenish nucleus has a sharp finely granular chromatin structure, and a well defined nuclear border.

The parabasal cells are slightly larger, round to oval in shape, and have brown fluorescent cytoplasm.

The intermediate (precornified) cells still retain slight traces of RNA and fluoresce a faint brown. On the nuclear membrane of the vesicular nucleus the sex chromatin or Barr body can be easily distinguished.

Superficial (cornified) squamous cells which contain very little or no RNA show pale green fluorescent cytoplasm. The nuclei are small, dense, green and pyknotic, because of degeneration and condensation of the chromatin material. It will be noted that the colour differentiation by the fluorescence method of the various types of squamous cells equals that of the Panicolaou method.

*Endocervical epithelium*

Cells originating from the columnar epithelium of the endocervical canal and the mucus producing glands are of two types. They are the columnar ciliated cells and the mucus producing secretory columnar cells. The latter fluoresce a reddish brown and contain yellowish eccentrically placed vesicular nuclei; nucleoli show a bright red. In well preserved columnar cells faint brown cilia are just visible at the terminal plate, unless cells are seen end on in honeycomb formation.

*Endometrial epithelium*

These cells occur mostly in tight clusters with greenish yellow nuclei which are difficult to resolve because of crowding and intensity of fluorescence. When present the cytoplasm fluoresces reddish brown.

*Histiocytes*

The cytoplasm of these cells shows a brown fluorescence; it may occasionally appear brilliant red when red fluorescent bacteria have been ingested. The eccentrically placed nuclei stain yellowish green, and show prominent nuclear borders and a rather coarse chromatin structure. They can be confused with basal or endometrial cells because of some similarities in size and structure. They are however easily identified by their typical morphological features, the often kidney shaped nuclei, and finely vacuolated cytoplasm. When they occur in groups, histiocytes have more tendency to spread out rather than to form clusters as often do endometrial cells. Multinucleated histiocytes are frequently observed, particularly in smears from post-menopausal patients.

*Polymorphs*

The lobulated nuclei of these cells fluoresce a bright green; the cytoplasm does not stain because of the absence of RNA. The nuclei may at times be surrounded by bright red bacteria that have been ingested into the cytoplasm. The polymorph is sometimes referred to as the cytologist's yardstick as its size is rather constant. Nuclear size of the polymorph can be compared against other structures as it is rarely absent from the microscope field.

*Red blood cells*

Erythrocytes remain invisible with the fluorescence technique and therefore do not interfere with the screening process; however, when interpreting some types of smears this may be a slight disadvantage.

*Bacteria*

Cocci and bacilli appear in bright red fluorescence ; they sometimes cover the surface of squamous cells. At first glance, this may be confusing to the eye, simulating cells with enhanced fluorescence. On close examination, however, the bacteria can readily be discerned adhering to the surface of the cells.

*Mucus*

This occurs in sheets and strands of a bright greenish yellow. When present in large quantities, it becomes troublesome as it produces considerable glare.

*Trichomonas vaginalis*

This infestation is easily recongised because of the bright red fluorescent often pear shaped cytoplasm of Trichomonas organisms ; the nuclei are a pale yellow located at one pole.

*Monilia albicans*

The mycelia and spores fluoresce a brilliant red and are readily recognisable, in fact more easily and frequently than with any other method.

*Physiological changes during the menstrual cycle, pregnancy and menopause.*

The phase of the menstrual cycle at which a particular smear has been collected is reflected in the cellular population, general architecture of cells and spread of the smear. Thus, upon examining a smear, it is possible to determine the approximate stage of the menstrual cycle at which the exfoliated material has been collected, or whether the patient is post-menopausal or pregnant.

A smear prepared from an aspiration taken from the posterior fornix of the vagina is most suitable for phasing. Scrapings from the portio of the cervix,

---

EXPLANATION OF PLATE

FIG. 1.—Scrape from normal cervix showing a group of basal and parabasal cells from the squamous epithelium with reddish brown cytoplasm and greenish yellow nuclei. Across the centre of the picture are several histiocytes with red fluorescent cytoplasm and yellow nuclei. Scattered over the field are bright green lobulated nuclei of polymorphs. (Female, 36) ×250.

FIG. 2.—Cervical scrape from a patient with a carcinoma *in situ* of the cervix. The group of cells show both fluorescent and morphological criteria for malignancy. (Female, 43) ×400.

FIG. 3.—Scrape from normal cervix. Intermediate and superficial squamous cells from a smear taken during the luteal phase. Scattered over the picture are bright green polymorphs and red Döderlein bacilli. (Female, 35) ×200.

FIG. 4.—Cervical scrape from a patient with an invasive squamous cell carcinoma of the cervix. The malignant cells have brilliant red fluorescent cytoplasm and intense yellow hyperchromatic nuclei. The cells also show all the morphological criteria for malignancy. (Female, 61) ×400.

FIG. 5.—Scrape from normal cervix. In the upper part of the picture a group of endocervical cells in honeycomb formation ; near the lower margin three individual columnar endocervical cells. The latter show reddish brown cytoplasm, the former more intense fluorescence because they are viewed end on through the whole length of the cells. (Female, 39) ×250.

FIG. 6.—Cervical scrape from a patient with invasive squamous cell carcinoma of the cervix. The malignant cells exhibit marked anisocytosis, anisokaryosis and bizarre cell shapes. Some show large nucleoli. (Female, 76) ×250.

1

2

3

4

5

6

Marks and Goodwin.

as these are routinely collected in this clinic, do perhaps not yield an entirely typical representation of the vaginal cell picture, particularly because of the traumatic effect produced by scraping. Nevertheless it is feasible also with such material to establish a consistent cellular pattern during the normal menstrual cycle. Endocervical and squamous epithelial cells, both from the superficial and deeper layers are removed upon scraping with a wooden spatula ; also more red and white blood cells than are typical for a particular point in the cycle may be added to the sample. Yet the presence of these additional cellular elements is of little concern during interpretation as with experience they are accepted as ordinary occurrence. Provided that the procedure of collecting and preparing exfoliated material is consistent, it is possible to establish a standard classification of vaginal smear types for the particular material and method of collection.

In this department, the endocrine status of all smears, whenever possible, is determined routinely. During fluorescence screening, the same morphological criteria as used on Papanicolaou stained smears, were applied. During screening the proportion of superficial squamous cells is estimated and compared with the expected level by checking it against the date of the patient's last menstrual period. Together with the other cell types present, and the general spread of the cells, we can confirm with a fair degree of accuracy that point of the cycle at which the exfoliated material was collected. Moreover, we know the proportion of polymorphs to expect at various points during the normal menstrual cycle ; any increase gives a fair indication as to the degree of infection present in the genital tract.

*Phases of the menstrual cycle*

*Menstrual phase* (days 1–4).—The large number of erythrocytes observed at this stage with Papanicolaou's method, are invisible with the fluorescence technique. Endometrial cells, singly and in groups, are scattered over the smear. Of the squamous cells, the brownish intermediate (precornified) cells are the more abundant ; the greenish superficial (cornified) cells make up about 10 to 20 per cent of all cells. The proportion of polymorphs is high. Abundant histiocytes are as a rule present. The bacterial flora is pronounced. Ample mucus, in degenerate, bright green strands occurs throughout the smear.

*Postmenstrual* or *proliferative phase* (days 5–11).—During this phase there is a gradual rise in the cornification curve until toward preovulation (days 11–13) green fluorescent superficial cells constitute about 30 to 40 per cent of all cells present. Thus in early stages the brownish fluorescent intermediate cells predominantly occur with yellowish green vesicular nuclei ; later more and more greenish superficial squamous cells with green pyknotic nuclei appear. With the AO method, the various cellular elements become clearer and more sharply defined with greater separation of the cells ; they appear in a much better state of preservation in this stage than at any other point in the cycle, except at ovulation. Basal cells, when present, often exhibit rather reddish cytoplasm, presumably because of their active proliferation in the phase. The proportion of polymorphs gradually diminishes towards midcycle. The bacterial flora, largely represented by Döderlein bacilli, are not very pronounced.

*Ovulatory phase* (day 14).—Coinciding with ovulation is a sharp increase in the percentage of superficial cornified squamous cells, that now constitute anywhere from 70 to 90 per cent of the total number of cells. With the fluorescence method, the superficial cells appear in pale translucent green with bright green pyknotic nuclei ; seen against a black background, the cells resemble rather the luminous face of a clock in a dark room. The cells are larger, with uniform transparent flattened and well spread abundant cytoplasm. Bacteria and polymorphs are scarce. Abundant greenish or greenish yellow mucus, forming strands and sheets, may occur.

*Luteal, progestational* or *secretory phase* (days 18–21).—Following ovulation the squamous cells begin to show progressive signs of degeneration ; there is a marked loss of transparency, the cytoplasm becomes granular and the margins often fold over. Cytolysis results in numerous dull green fluorescent free nuclei. The green superficial cells gradually decrease in number and the intermediate cell becomes the predominant cell type. The squamous cells may be clumped to form smaller and larger aggregates with ochre brown fluorescence that, because of diminished contrast, renders nuclei rather pale. During the first part of this phase the proportion of polymorphs is still low. Toward the menstrual phase it gradually increases, together with reddish brown or red fluorescent Döderlein bacilli, that may occur in enormous numbers particularly during the latter part of the secretory phase. In instances, bacilli cling in great numbers to the surface of squamous cells so making them appear to have increased fluorescence.

The phasing of smears with the AO method is both accurate and simple. The point of ovulation and menstruation is readily determined cytologically ; the degree of transparency of the cytoplasm of the squamous cells present will fit the smear into either of the phases preceding or following ovulation. Poor transparency will place the smear into the progestational phase, whereas a clear and sharp picture, with a good degree of cytoplasmic transparency will fit it into the postmenstrual to ovulatory phase. With practice, the degrees of changes, the general architecture of the smear, the cell types present and their proportions will enable the cytologist to pinpoint with accuracy to within three to four days the point of the cycle at which the exfoliated material has been collected. If the date of the onset of the last normal menstrual period was ascertained when the aspiration was taken, then any departure from the expected cytological picture will alert the cytologist to abnormality that may be present.

*Pregnancy* can often be recognised by the predominance of brownish fluorescent intermediate cells with large, vesicular greenish fluorescent nuclei. These cells are frequently clumped together to form large irregular sheets or aggregates ; the cells have irregular cytoplasmic edges and show folding. Occasionally groups of cells with thickened cellular edges can be observed. With progressive pregnancy increased cytolysis occurs, resulting in numerous green fluorescent, free nuclei. Some of the intermediate cells may assume roughly ellipsoidal shape, and are then referred to as " navicular cells " ; such are not present in all pregnancy smears, however, and may occur also in exfoliated material from patients under excess progestational influence. Döderlein bacilli are usually abundant and often form a background all over the smear of reddish brown or red fluorescent, regular shaped rods. The proportion of polymorphs is normally rather moderate.

*Menopausal and postmenopausal* smears show a deficiency of mature squamous cells, particularly those of the superficial variety. Early in the menopause, intermediate cells predominate. In later stages, a mixture of intermediate squamous and parabasal cells occurs, and smears are devoid of superficial squamous cells. The typical postmenopausal " atrophic " smear contains predominantly cells resembling the parabasal type ; sometimes these cells have enlarged nuclei. Occasional basal cells may likewise occur. The cytoplasm of normal parabasal cells is brownish ; in atrophic smears parabasal cells may show increased cytoplasmic fluorescence, which sometimes may be brilliant orange. The green or greenish yellow fluorescent nuclei may be large and show signs of degeneration, such as pyknosis and karyorrhexis. In some smears, bare nuclei of parabasal cells may be abundant. The proportion of polymorphs may be rather high in cases with some degree of vaginitis or cervicitis, that may occur rather frequently ; particularly in chronic cases, histiocytes may then also be present.

Caution is advisable not to regard as suspicious some postmenopausal smears of the atrophic type, particularly from patients with senile vaginitis ; such smears may contain basal and parabasal cells with bright orange fluorescence, that have become shed individually or in large sheets. Examination of the nuclei will determine whether the cells are normal or atypical.

*Erosion* of the cervix is usually evident by greatly increased numbers of endocervical cells. With this condition endocervical cells presumably proliferate at faster rates and consequently contain larger amounts of RNA (L. von Bertalanffy, F. D. Bertalanffy and Goodwin, 1961). They may thus show bright red cytoplasmic fluorescence ; care should be taken not to mistake them for suspicious cells. Study of their morphology will readily reveal their normal structure.

*Trichomonas* infestation and *cervicitis* sometimes give rise to bright red, active basal cells ; the nuclei may be enlarged, show a prominent border and increased fluorescence. Examination of the nuclei usually reveals that the chromatin has become concentrated around the periphery of the nucleus at its border, whilst leaving a few prominent clumps of chromatin in the centre. Other indications of the Trichomonas infestation is the concentration of bright green polymorphs on the surface of some squamous cells. Usually the smear has a background consisting of finely granular, brownish red debris. Frequently associated with this infestation are the red fluorescent long hair-like filaments of leptothrix bacilli.

*Cellular atypia* associated with *pregnancy* might occasionally give rise to some difficulties in interpretation. Atypicalities sometimes occur in cells from the more superficial layers that show a slight increase in RNA. The nuclei may be enlarged and irregular but the nuclear-cytoplasmic ratio is usually within normal limits ; these atypical cells occasionally show binucleation. Patients with such cellular changes have their cytosmears repeated until the atypical cells disappear or become definitely suspicious. During the present series, carcinoma *in situ* was detected in three patients with such smears ; our overall incidence of cases with carcinoma *in situ* among the obstetrical patients is 0·5 per cent (Goodwin and Marks, 1962).

*Suspicious and malignant cells*, as with any cytodiagnostic procedure, have to be examined closely as to their morphological structure to ascertain whether or not they fulfill any of the criteria for malignancy. In the authors' experience it is somewhat easier to recognise atypical cells with the AO method but a little more difficult to interpret cells that fall into the dyskaryotic category. However, it is the detection of the abnormal cells that is the primary consideration. Repeat smears of doubtful cases will usually reveal the true nature of the cellular changes. With the fluorescence method, the majority of malignant cells exhibit most striking red or orange fluorescent cytoplasm. The nuclei show green fluorescence or greenish yellow particularly when smaller and hyperchromatic. Degenerating cells, as such may occur among the bright red fluorescent cancer cells, show gradual diminution of cytoplasmic fluorescence that may be a reddish brown ; the nuclear structure and fluorescence usually is still preserved, even though signs of degeneration may be apparent also in the nuclei of such cells.

It must be remembered that acridine orange is not a specific stain for malignant cells. This has mistakenly been assumed by some who then have unjustly criticised the method as being unreliable. If the morphological criteria for the identification of cells with increased fluorescence are observed, their normal or abnormal structure can be identified correctly, and very few false positive interpretations will occur. The AO technique has been developed primarily for the cytochemical demonstration of RNA and DNA, in which the rapidly proliferating malignant cells are especially rich. It is thus indeed surprising that it also reveals as much morphological detail, sufficient for final cytodiagnosis. For what little may be lost is outweighed by the additional pointer to suspicious and malignant cells that appear in the most striking fluorescence colours of red (RNA) and green or yellow (DNA).

### CONCLUSIONS

Screening with the fluorescence method is both rapid and easy, and there is less necessity to concentrate on cellular structure as abnormal cells give themselves away by virtue of their most brilliant colour. It is recommended that cytology laboratories that are experiencing difficulties in dealing with the volume of work with their existing facilities should seriously consider making use of the AO method as it has the advantages of requiring less time for the preparation and screening of smears. The technique lends itself to prescreening by personnel with less cytological experience (Connally and Wall, 1960 ; F. D. Bertalanffy 1961*c*), but final diagnoses of malignancy must be made by the experienced and competent cytologist.

### SUMMARY

The application of the acridine orange fluorescence method for cytodiagnosis of exfoliated material from the female genital tract is discussed, based upon the experience obtained whilst screening with this method routine smears from over 4500 gynaecological and obstetric patients. Detailed descriptions of normal and abnormal cells, as they appear with the fluorescence technique, are supplied. Particularly the procedure of ascertaining the endocrine status of the patient by the acridine orange stained smear is explained. Determination of the phase of the menstrual cycle, pregnancy, and menopause has been found as readily feasible by the fluorescence as by the Papanicolaou method. Attention has been called to some circumstances where false interpretations could occur if sufficient care is not taken.

It is concluded that the fluorescence technique is faster and simpler (requiring only 7 minutes for the preparation of smears), and expedites the screening process. Fluorescence cytodiagnosis can be applied in place of existing techniques of exfoliative cytology without loss in accuracy, and is particularly recommended for use in cytology laboratories experiencing difficulties in dealing with the volume of smears to be processed.

The authors wish to thank Dr. F. D. Bertalanffy, in whose laboratory this investigation was carried out, for his help in the preparation of this manuscript. We also gratefully acknowledge the kind cooperation of Dr. W. J. Friesen and Dr. C. C. Henneberg, Manitoba Clinic, who supplied part of the exfoliative material.

This work was supported by a research grant from the National Cancer Institute of Canada, Toronto.

### REFERENCES

Armstrong, J. A.—(1956) *Exp. Cell. Res.*, **11**, 640.

Bertalanffy, F. D.—(1960*a*) *CA*, **10**, 118.—(1960*b*) *Postgrad. Med.*, **28**, 627.—(1960*c*) *Mikroskopie*, **15**, 67.—(1961*a*) *Cancer Res.*, **21**, 422.—(1961*b*) *Krebsarzt*, **16**, 521.—(1961*c*) *Canad. J. med. Technol.*, **23**, 153.

von Bertalanffy, L.—(1959) *Klin. Wschr.*, **37**, 469.

*Idem* and Bertalanffy, F. D.—(1960) *Ann. N. Y. Acad. Sci.*, **84**, 225.—(1961) *Med. Welt*, **35**, 1742.

*Idem*, Bertalanffy, F. D. and Goodwin, A. M.—(1961) *Acta Cytol.*, **5**, 256.

*Idem* and Bickis, I.—(1956) *J. Histochem. Cytochem.*, **4**, 481.

*Idem*, Masin, F. and Masin, M.—(1956) *Science*, **124**, 1024.—(1958) *Cancer*, **11**, 873.

Bontke, E., Kern, G. and Schümmelfeder, N.—(1960) *Geburtsh. u. Frauenheilk.* **20**, 24.

Connally, H. F., Jr. and Wall, J. A.—(1960) *Tex. St. J. Med.*, **56**, 846.

Dart, L. H., Jr. and Turner, T. R.—(1959) *Lab. Invest.*, **8**, 1513.

Dubrauszky, V.—(1961) *Zbl. Gynäk.*, **83**, 1396.

Elevitch, F. R. and Bruson, J. G.—(1961) *Surg. Gynec. Obstet.*, **112**, 3.

Goodwin, A. M. and Marks, R.—(1962) *Manitoba med. Ass. Rev.*, **42**, 210.

Grubb, C. and Crabbe, J. G. S.—(1961) *Brit. J. Cancer*, **15**, 483.

Hitchcock, C. R. and Scheiner, S. L.—(1961) *Surg. Gynec. Obstet.*, **113**, 665.

Holland, J. C. and Ackermann, M. R.—(1961) *Obstet. Gynec.*, **17**, 38.

Hunter, D. T., Brown, D. N. and Redmond, R. F.—(1959) *J. Okla. med. Ass.*, **52**, 772.

Kaplan, L., Masin, F., Masin, M., Carleton, R. and von Bertalanffy L.—(1960) *Amer. J. Obstet. Gynec.*, **80**, 1063.

Monter, H. and Navarro, G.—(1961) *Rev. med. Hosp. gen. Méx.*, **24**, 237.

van Niekerk, W. A.—(1960) *S. Afr. Cancer Bull.*, **4**, 185.

Schümmelfeder, N., Ebschner, K. J. and Krogh, E.—(1957) *Naturwissenschaften*, **44**, 467.

Sussman, W.—(1959) *Obstet. Gynec.*, **13**, 273.—(1961) *Amer. J. Obstet. Gynec.*, **82**, 1272

Umiker, W.—(1961) *Acta Cytol.*, **5**, 245.

*Idem* and Pickle, L.—(1960) *Lab. Invest.*, **9**, 613.

*Iidem* and Waite, B.—(1959) *Brit. J. Cancer*, **13**, 398.

Zanella, E. and Chiampo, L.—(1961) *Boll. Soc. ital. Biol. Sper.* **37**, 652.